# Some New Algebraic and Topological Properties of the Minkowski Inverse in the Minkowski Space

**DOI:** 10.1155/2013/765732

**Published:** 2013-10-29

**Authors:** Hanifa Zekraoui, Zeyad Al-Zhour, Cenap Özel

**Affiliations:** ^1^Department of Mathematics and Informatic, Faculty of Exact and Natural Sciences, Oum-El-Bouaghi University, Oum-El-Bouaghi 04000, Algeria; ^2^Department of Basic Sciences and Humanities, College of Engineering, University of Dammam, P.O. Box 1982, Dammam 34151, Saudi Arabia; ^3^Department of Mathematics, Faculty of Sciences, University of Abant Izzet Baysal, Bolu Turkey, 14280 Bolu, Turkey

## Abstract

We introduce some new algebraic and topological properties of the Minkowski inverse *A* ^⊕^ of an arbitrary matrix *A* ∈ *M*_*m*,*n*_ (including singular and rectangular) in a Minkowski space *μ*. Furthermore, we show that the Minkowski inverse *A* ^⊕^ in a Minkowski space and the Moore-Penrose inverse *A* ^+^ in a Hilbert space are different in many properties such as the existence, continuity, norm, and SVD. New conditions of the Minkowski inverse are also given. These conditions are related to the existence, continuity, and reverse order law. Finally, a new representation of the Minkowski inverse *A* ^⊕^ is also derived.

## 1. Introduction and Preliminaries

In this work, we consider matrices over the field of complex numbers as *ℂ* and real numbers as ℝ. The set of *m*-by-*n* complex matrices is denoted by *M*_*m*,*n*_(*ℂ*) = *ℂ* ^*m*×*n*^. For simplicity, we write *M*_*m*,*n*_ instead of *M*_*m*,*n*_(*ℂ*) or *M*_*m*,*n*_(ℝ), and when *m* = *n*, we write *M*_*n*_ instead of *M*_*n*,*n*_. The notations *A* ^*t*^, A*, *A* ^~^,  *r*(*A*), *R*(*A*), *N*(*A*), tr⁡(*A*), det⁡(*A*), ||*A*||_2_, *A* ^+^, *A* ^⊕^, and *σ*(*A*) stand for the transpose, conjugate transpose, *μ*-symmetric, rank, range, null space, trace, determinant, Frobenius norm, Moore-Penrose inverse, Minkowski inverse, and set of all eigenvalues of a matrix *A*, respectively.

The Moore-Penrose inverse is widely used in perturbation theory, singular systems, neural network problems, least-squares problems, optimization problems, and many other subjects [1–8]. The Moore-Penrose inverse of an arbitrary matrix *A* ∈ *M*_*m*,*n*_ is defined to be the unique solution of the following four matrix equations [3, 4, 8–10]:

(1) $$\begin{array}{c} AXA=A,\;\;XAX=X,\\ {\left(AX\right)}^{*}=AX,\;\;{\left(XA\right)}^{*}=XA, \end{array}$$

and it is often denoted by *X* = *A* ^+^. Note that if we designate any matrix *X* that satisfying the *i*th matrix equation (*i* ∈ {1,2, 3,4}) in (1) is called the *i*-inverse and denoted by *A* ^(*i*)^.

The Moore-Penrose inverse can be explicitly expressed by the singular value decomposition (SVD) due to van Loan [11]. For any matrix *A* ∈ *M*_*m*,*n*_ with *r*(*A*) = *r*, there exist unitary matrices *U* ∈ *M*_*m*_ and *V* ∈ *M*_*n*_ satisfying U*U = *I*_*m*_ and V*V = *I*_*n*_ such that

(2) $$\begin{array}{c} A=U\left[\begin{array}{cc} D & 0\\ 0 & 0 \end{array}\right]V^{*}, \end{array}$$

where *D* = diag⁡(*δ*_1_, *δ*_2_,…, *δ*_*r*_) ∈ *M*_*r*_, *δ*_1_ ≥ *δ*_2_ ≥ ⋯≥*δ*_*r*_ > 0, and *δ*_*i*_^2^ (*i* = 1,2,…, *r*) are the nonzero eigenvalues of A*A. Then, the Moore-Penrose inverse can be represented as

(3) $$\begin{array}{c} A^{+}=V\left[\begin{array}{cc} D^{-1} & 0\\ 0 & 0 \end{array}\right]U^{*}. \end{array}$$

Some algebraic properties concerning the null space, range, rank, continuity, and some representations of some types of the generalized inverses of a given matrix over complex and real fields are widely studied by many researchers [12–16]. The Minkowski inverse *A* ^⊕^ of an arbitrary matrix *A* ∈ *M*_*m*,*n*_ is one of the important generalized inverses for solving matrix equations in the Minkowski space *μ* with respect to the generalized reflection antisymmetric matrix *A* ^~^ [17]. Some methods such as iterative, Borel summable, Euler-Knopp summable, Newton-Raphson, and Tikhonov's methods are used for representation and computation of the Minkowski inverse *A* ^⊕^ in the Minkowski space *μ* [18, 19].

By letting *ℂ* ^*n*^ be the space of complex *n*-tuples, we will index the components of a complex vector in *ℂ* ^*n*^ from 0 to *n* − 1; that is, *u* = (*u*_0_, *u*_1_, *u*_2_,…, *u*_*n*−1_). In addition to that, let *G* be the Minkowski metric tensor defined by *Gu* = (*u*_0_, −*u*_1_, −*u*_2_,…, −*u*_*n*−1_). Clearly, the Minkowski metric matrix is defined by [18, 20]

(4) $$\begin{array}{c} G=\left[\begin{array}{cc} 1 & 0\\ 0 & -I_{n-1} \end{array}\right]\in M_{n}, \end{array}$$

and G* = *G* and *G* ^2^ = *I*_*n*_.

In [21, 22], the Minkowski inner product on *ℂ* ^*n*^ is defined by (*u*, *v*) = [*u*, *Gv*], where [·, ·] denotes the conventional Hilbert (unitary) space inner product. The space with the Minkowski inner product is called a Minkowski space and is denoted by *μ*. For any square matrix *A* ∈ *M*_*n*_ and vectors *x* and *y* ∈ *ℂ* ^*n*^, we have

(5)   (Ax,y)=[Ax,Gy]=[x,A∗Gy]=[x,G(GA∗G)y]=[x,GA~y]=(x,A~y),

where *A* ^~^ = GA*G is called the Minkowski conjugate transpose of *A* in the Minkowski space *μ*. Naturally, the matrix *A* ∈ *M*_*n*_ is called *μ*-symmetric in the Minkowski space *μ* if *A* = *A* ^~^. Now, it is easy to show that *A* is *μ*-symmetric if and only if *AG* is Hermitian if and only if *GA* is Hermitian. Also, it is easy to verify that *G* ^−1^ = *G* and *σ*(*A* ^~^) = *σ*(*A*). More generally, if *A* ∈ *M*_*m*,*n*_, then the Minkowski conjugate transpose of *A* is defined by *A* ^~^ = *G*_1_ A*G_2_ (where  *G*_1_ and *G*_2_ are the Minkowski metric matrices of orders *n* × *n* and *m* × *m*, resp.), and it satisfies the following algebraic properties as in the following result.

**Lemma 1**

Let *A* ∈ *M*_*m*,*n*_. Then, the following one given:

- *A* ^~^ is unique,
- (*A* ^~^)^~^ = *A*,
- (*AB*)^~^ = *B* ^~^ *A* ^~^,
- ~-cancellation rule *A* ^~^ *AX* = *A* ^~^ *AY*⇒*AX* = *AY*,
- *r*(*A*) = *r*(*A* ^~^),
- *R*(A*) = *R*(*A* ^~^),
- *N*(A*) = *N*(*A* ^~^).

Finally, a matrix *A* ∈ *M*_*m*,*n*_ is said to be a range symmetric in unitary space (or equivalently *A* is said to be EP) if *N*(A*) = *N*(*A*). For further properties of EP matrices, one may refer to [3, 4, 10, 11].

In this paper, some algebraic properties concerning the rank, range, existence, uniqueness, continuity, and reverse order law of the Minkowski inverse *A* ^⊕^ are introduced. The relationships between *A* ^⊕^ and *A* ^~^ are also discussed. Furthermore, a new representation of *A* ^⊕^ related to the full-rank factorization of the matrix *A* is derived, and new conditions for the existence and continuity of *A* ^⊕^ are also given.

## 2. Some Algebraic Properties of the Minkowski Inverse

In this section, we derive some attractive algebraic properties and the reverse order law property of the Minkowski inverse in a Minkowski space.

The Minkowski inverse of an arbitrary matrix *A* ∈ *M*_*m*,*n*_ (including singular and rectangular), analogous to the Moore-Penrose inverse, is defined as follows.

**Definition 2**

Let *A* ∈ *M*_*m*,*n*_ be any matrix in the Minkowski space *μ*. Then, the Minkowski inverse of *A* is the matrix *A* ^⊕^ ∈ *M*_*n*,*m*_ which satisfies the following four matrix equations:

(6) $$\begin{array}{c} AA^{⊕}A=A,\;\;A^{⊕}AA^{⊕}=A^{⊕},\\ {\left(AA^{⊕}\right)}^{\sim }=AA^{⊕},\;\;{\left(A^{⊕}A\right)}^{\sim }=A^{⊕}A. \end{array}$$

**Theorem 3**

Let *A* ∈ *M*_*m*,*n*_ be any matrix in the Minkowski space *μ*. Then, the Minkowski inverse *A* ^⊕^ satisfies the following properties:

- (*A* ^⊕^)^~^ = (*A* ^~^)^⊕^,
- *A* ^⊕^ is a unique matrix,
- (*A* ^⊕^)^⊕^ = *A*,
- *AA* ^⊕^ and *A* ^⊕^ *A* are idempotents (i.e., *AA* ^⊕^ and *A* ^⊕^ *A* are projectors on *R*(*A*) and *R*(*A* ^⊕^), resp.),
- *A* ^⊕^ *A* + *αI*_*n*_ and *AA* ^⊕^ + *αI*_*m*_ are invertible matrices, where *α* > 0,
- (*A* ^~^ *A*)^⊕^ *A* ^~^ = *A* ^⊕^ = *A* ^~^(*AA* ^~^)^⊕^,
- *R*(*A* ^⊕^) = *R*(*A* ^~^).

**Proof**

(i)Since the following four matrix equations are satisfied:

(7) A~(A⊕)~A~=(AA⊕A)~=A~,(A⊕)~A~(A⊕)~=(A⊕AA⊕)~=(A⊕)~,(A~(A⊕)~)~=((A⊕)~)~(A~)~=A⊕A=(A⊕A)~=A~(A⊕)~,((A⊕)~A~)~=((A~)~)((A⊕)~)~=AA⊕=(AA⊕)~=(A⊕)~A~,

then, by (6), we get the result.

(ii)Let *G*_1_ and *G*_2_ be two Minkowski metric tensors such that *A*_1_^⊕^ and *A*_2_^⊕^ are two Minkowski inverses of a matrix *A*; then, by using Lemma 1 and Theorem 3(i), we have

(8) A1⊕=A1⊕AA1⊕=A1⊕(AA1⊕)~=A1⊕(A1⊕)~A~=A1⊕(A1⊕)~A~(A2⊕)~A~=A1⊕(AA1⊕)~(AA2⊕)~=A1⊕(AA1⊕)(AA2⊕)=A1⊕AA2⊕=A1⊕AA2⊕AA2⊕=(A1⊕A)~(A2⊕A)~A2⊕=A~(A1⊕)~A~(A2⊕)~A2⊕=A~(A2⊕)~A2⊕=(A2⊕A)~A2⊕=(A2⊕A)A2⊕=A2⊕.

This means that *A* ^⊕^ is a unique matrix.

(iii) It follows by applying the four matrix equations in (6).

(iv) By using the matrix equations in (6), we have (*AA* ^⊕^)^2^ = *A*(*A* ^⊕^ *AA* ^⊕^) = *AA* ^⊕^ and (*A* ^⊕^ *A*)^2^ = *A* ^⊕^(*AA* ^⊕^ *A*) = *A* ^⊕^ *A*.

(v) Since *AA* ^⊕^ is an idempotent matrix, then eigenvalues of *AA* ^⊕^ are 0 or 1. That is, det⁡(*AA* ^⊕^ + *αI*_*m*_) = 0⇔*α* = 0 or *α* = −1. So, for all *α* > 0, we have det⁡(*AA* ^⊕^ + *αI*_*m*_) ≠ 0 (i.e., *A* ^⊕^ *A* + *αI*_*n*_  is an invertible matrix). Similarly, we can prove that *AA* ^⊕^ + *αI*_*m*_ is also an invertible matrix.

(vi) Since *A* ^~^ *A*(*A* ^~^ *A*)^⊕^ *A* ^~^ *A* = *A* ^~^ *A*, then, from ~-cancellation, we have

(9) $$\begin{array}{c} A{\left(A^{\sim }A\right)}^{⊕}A^{\sim }A=A. \end{array}$$

Now, by using the four matrix equations in (6), Theorem 3, and Lemma 1, we have

(10) ((A~A)⊕A~)A((A~A)⊕A~)=((A~A)⊕(A~A)(A~A)⊕)A~=(A~A)⊕A~,

(11) (A(A~A)⊕A~)~=A((A~A)⊕)~A~=A((A~A)~)⊕A~=A(A~A)⊕A~,

(12) ((A~A)⊕A~A)~=A~A(A~A)⊕=(A~A(A~A)⊕)~=(A~A)⊕A~A.

Consequently, (9), (10), and (11) show that (*A* ^~^ *A*)^⊕^ *A* ^~^ = *A* ^⊕^.

(vii) Equations (9) and (10) show that *r*(*A* ^⊕^) = *r*(*A*) = *r*(*A* ^~^). Now, by applying Theorem 3(vi), we have *R*(*A* ^⊕^) ⊂ *R*(*A* ^~^); then, the equality holds.

The reverse order law property for the Moore-Penrose inverse of the product of two matrices is investigated by many researchers; one may refer to [23]. Analogous to Greville's conditions that were stated in [6], we reached the following result.

**Theorem 4**

Let *A* ∈ *M*_*m*,*n*_ and *B* ∈ *M*_*n*.*p*_ be two matrices in the Minkowski space *μ* such that the Minkowski inverses *A* ^⊕^, *B* ^⊕^, and (*AB*)^⊕^ exist. Then, (*AB*)^⊕^ = *B* ^⊕^ *A* ^⊕^ if and only if *R*(*A* ^~^ *AB*) ⊂ *R*(*B*) and *R*(*BB* ^~^ *A* ^~^) ⊂ *R*(*A* ^~^).

**Proof**

Since *BB* ^⊕^ is a projector on *R*(*B*) as in Theorem 3(iv), then

(13) $$\begin{array}{c} BB^{⊕}A^{\sim }AB=A^{\sim }AB. \end{array}$$

Now, by Definition 2 and Theorem 3, we have

(14) $$\begin{array}{c} A^{⊕}ABB^{\sim }A^{\sim }=BB^{\sim }A^{\sim }. \end{array}$$

Taking the Minkowski conjugate transpose of the two sides of (13), we have

(15) $$\begin{array}{c} B^{\sim }A^{\sim }ABB^{⊕}=B^{\sim }A^{\sim }A. \end{array}$$

Multiplying the right side and the left side of (15) by *A* ^⊕^ and ((*AB*)^~^)^⊕^, respectively, we have

(16) $$\begin{array}{c} {\left({\left(AB\right)}^{\sim }\right)}^{⊕}{\left(AB\right)}^{\sim }\left(AB\right)B^{⊕}A^{⊕}={\left({\left(AB\right)}^{\sim }\right)}^{⊕}{\left(AB\right)}^{\sim }AA^{⊕}. \end{array}$$

Since *R*(*AB*) ⊂ *R*(*A*) = *R*(*AA* ^⊕^), then we have

(17) ABB⊕A⊕=((AB)(AB)⊕)~(AB)B⊕A⊕=((AB)(AB)⊕)~AA⊕=(AB)(AB)⊕.

Also, multiplying the right side and the left side of (14) by ((*AB*)^~^)^⊕^ and *B* ^⊕^, respectively, and applying Theorem 3 and Definition 2 for (*AB*)^⊕^, we have

(18) $$\begin{array}{c} B^{⊕}A^{⊕}AB{\left(AB\right)}^{⊕}\left(AB\right)=B^{⊕}BB^{\sim }A^{\sim }{\left({\left(AB\right)}^{\sim }\right)}^{⊕}. \end{array}$$

Since *B* ^⊕^ *B* is a projector on *R*(*B* ^~^), we have

(19) $$\begin{array}{c} B^{⊕}A^{⊕}AB=B^{\sim }A^{\sim }{\left({\left(AB\right)}^{\sim }\right)}^{⊕}={\left(\left(AB\right)\right)}^{⊕}\left(AB\right). \end{array}$$

Equations (17) and (19) imply that *B* ^⊕^ *A* ^⊕^ satisfies the first, third, and fourth equations in (6). Finally, by taking the Minkowski conjugate transpose of the two sides of the first and the second equations in (6) for matrices *A* and *B* and by using Theorem 3(vi), we have

(20) $$\begin{array}{c} B^{\sim }A^{\sim }=B^{\sim }BB^{⊕}A^{⊕}AA^{\sim },\\ B^{⊕}A^{⊕}=B^{⊕}{\left(B^{⊕}\right)}^{\sim }B^{\sim }A^{\sim }{\left(AA^{\sim }\right)}^{⊕}. \end{array}$$

This equation shows that

(21) $$\begin{array}{c} r\left(B^{⊕}A^{⊕}\right)=r\left(B^{\sim }A^{\sim }\right)=r{\left(AB\right)}^{\sim }=r\left(AB\right). \end{array}$$

Consequently, *B* ^⊕^ *A* ^⊕^ satisfies the second equation in (6).

## 3. Existence of the Minkowski Inverse

The Minkowski inverse of a matrix *A* exists if and only if *r*(*AA* ^~^) = *r*(*A* ^~^ *A*) = *r*(*A*) [12]. In this section, we give some equivalent conditions for the existence and derive a new representation of the Minkowski inverse. If *A* ∈ *M*_*m*,*n*_ is a matrix of full row rank (column rank), then AA* and A*A are invertible matrices of orders *m* × *m* and *n* × *n*, respectively, in a Hilbert (Euclidian) space. Here, in a Minkowski space, if we define ||*A*||_02_ = (tr⁡(*AA* ^~^))^1/2^, then the following example shows that *A* ^~^ *A* and *AA* ^~^ are, in general, not invertible matrices and also ||*A*||_02_ ≠ ||*A*||_2_.

**Example 5**

Let $A=\left[\begin{array}{ccc} 1 & -1 & 1\\ 1 & 1 & 1 \end{array}\right]$. Then, *r*(*A*) = 2, and

(22) A~=G1A∗G2=[1000−1000−1][11−1111][100−1]=[1−111−11],AA~=[1−11111][1−111−11]=[−1−111].

Note that *r*(*AA* ^~^) = 1 (i.e., det⁡(*AA* ^~^) = 0), and hence *AA* ^~^ is not invertible. Also, ${||A||}_{2}=\sqrt{\mathrm{tr}(AA^{*})}=\sqrt{6}$, and ||*A*||_02_ = 0, which are not equal.

**Lemma 6**

Let *A* ∈ *M*_*m*,*n*_ and *B* ∈ *M*_*n*.*p*_ be two matrices. Then, the following are considered.

- If *r*(*A*) = *n*, then *r*(*AB*) = *r*(*B*).
- If *r*(*B*) = *n*, then *r*(*AB*) = *r*(*A*).

**Proof**

(i) Since *r*(*A*) = *n*, then *A* is a left invertible; thus, there exists a matrix *X* ∈ *M*_*n*,*m*_ such that *XA* = *I*_*n*_. Hence, *r*(*B*) = *r*(*X* *AB*) ≤ *r*(*AB*) ≤ *r*(*B*), which implies that *r*(*AB*) = *r*(*B*).  Similarly, we can prove (ii).

**Theorem 7**

Let *A* = *BC* ∈ *M*_*m*,*n*_ be a rank factorization of rank *r*. Then, (*CC* ^~^)^−1^ and (*B* ^~^ *B*)^−1^ exist if and only if *r*(*AA* ^~^) = *r*(*A* ^~^ *A*) = *r*(*A*).

**Proof**

Since *A* = *BC*, then *B* and *C* are of orders *m* × *r* and *r* × *n*, respectively, and *r*(*A*) = *r*(*B*) = *r*(*C*) = *r*. Hence,

(23) $$\begin{array}{c} AA^{\sim }=BC\left(G_{1}C^{*}G\right)\left(GB^{*}G_{2}\right)=BCC^{\sim }B^{\sim }, \end{array}$$

where *G*, *G*_1_, and *G*_2_ are the Minkowski metric matrices of orders *r*  ×  *r*, *n*  ×  *n*, and *m* × *m*, respectively. Since *B* and *B* ^~^ are matrices of full ranks (i.e., *r*(*B*) = *r*(*B* ^~^) = *r*), then, by Lemma 6, we have *r*(*AA* ^~^) = *r*(*CC* ^~^). Similarly, we can prove that *r*(*A* ^~^ *A*) = *r*(*B* ^~^ *B*).

Now, since *B* ^~^ *B* and *CC* ^~^ are square matrices of order *r*  ×  *r*, then they are invertible if and only if they are of rank *r*.

By applying the four matrix equations in (6), we can get a new representation of the Minkowski inverse as shown in the following result.

**Theorem 8**

Let *A* = *BC* ∈ *M*_*m*,*n*_ be a rank factorization of rank *r*. Then,

(24) $$\begin{array}{c} A^{⊕}=C^{\sim }{\left(CC^{\sim }\right)}^{-1}{\left(B^{\sim }B\right)}^{-1}B^{\sim }. \end{array}$$

## 4. Some Topological Properties of the Minkowski Inverse

In this section, we establish some attractive topological properties and new conditions for the continuity of the Minkowski inverse in a Minkowski space.

It is known that, in normed algebra of bounded linear operators, the map of linear invertible operators associated with its usual inverse is continuous. The following example shows that this property is not valid in the Minkowski space.

**Example 9**

Let $A_{n}=\left[\begin{array}{cc} 1 & 0\\ 0 & 1/n \end{array}\right]$ be a sequence of matrices for *n* ∈ *ℕ*; then, $A_{n}^{⊕}=\left[\begin{array}{cc} 1 & 0\\ 0 & n \end{array}\right]$, $\lim_{n\to \infty }A_{n}=\left[\begin{array}{cc} 1 & 0\\ 0 & 0 \end{array}\right]$, and ${\left(\lim_{n\to \infty }A_{n}\right)}^{⊕}=\left[\begin{array}{cc} 1 & 0\\ 0 & 0 \end{array}\right]$. Note that lim⁡_*n*→*∞*_ *A*_*n*_^⊕^ does not exist. That is, for the map *T*, we have

(25) $$\begin{array}{c} T\left(\underset{n\to \infty }{\lim}A_{n}\right)\neq \underset{n\to \infty }{\lim}T\left(A_{n}\right). \end{array}$$

For ||*A* ^⊕^||_02_ ≠ 0, the following results are very important for finding the new conditions for the continuity of the Minkowski inverse of rectangular matrices in a Minkowski space.

**Lemma 10**

Let *A* ∈ *M*_*m*,*n*_. Then, for any *x* ∈ *R*(*A* ^~^),

(26) $$\begin{array}{c} {||Ax||}_{02}\geq \frac{{||x||}_{02}}{{||A^{⊕}||}_{02}}. \end{array}$$

**Proof**

By using Theorem 3((iv) and (vii)), then, for any *x* ∈ *R*(*A* ^~^), we have *x* = *A* ^⊕^ *Ax*. Thus, ||*x*||_02_ = ||*A* ^⊕^ *Ax*||_02_ ≤ ||*A* ^⊕^||_02_||*Ax*||_02_, and then we get the result.

**Lemma 11**

Let *A* and *E* ∈ *M*_*m*,*n*_ such that  ||*A* ^⊕^||_02_ ≠ 0 and ||*E*||_02_ < 1/||*A* ^⊕^||_02_. Then,

(27) $$\begin{array}{c} r\left(A+E\right)\geq r\left(A\right). \end{array}$$

**Proof**

Suppose that *r*(*A*) = *r* and {*v*_1_,…, *v*_*r*_} are the basis of *R*(*A* ^~^). Then, the set {(*A* + *E*)(*v*_1_),…, (*A* + *E*)(*v*_*r*_)} is a subset of *R*(*A* + *E*). Now, suppose that ∑_*i*=1_^*r*^ *α*_*i*_(*A* + *E*)(*v*_*i*_) = 0, for *α*_*i*_ ∈ *ℂ*; then, *x* = ∑_*i*=1_^*r*^ *α*_*i*_ *v*_*i*_ ≠ 0, and we have

(28) 0=||∑ri=1αi(A+E)(vi)||02=||(A+E)∑ri=1αivi||02=||Ax+Ex||02≥||Ax||02−||E||02||x||02>||Ax||02−||x||02||A⊕||02.

Now, by using Lemma 10, we have 0 > (||*x*||_02_/||*A* ^⊕^||_02_) − (||*x*||_02_/||*A* ^⊕^||_02_) = 0, which is impossible, and thus *x* = 0. As {*v*_1_,…, *v*_*r*_} is linearly independent, it follows that *α*_*i*_ = 0 (for all *i* = 1,…, *r*). Consequently, *r*(*A* + *E*) ≥ *r* = *r*(*A*).

**Corollary 12**

Let *A* and *B* ∈ *M*_*m*,*n*_ such that ||*A* ^⊕^||_02_ ≠ 0,  ||*B* ^⊕^||_02_ ≠ 0, and ||*A*−*B*||_02_ < 1/max⁡{||*A* ^⊕^||_02_, ||*B* ^⊕^||_02_}. Then,

(29) $$\begin{array}{c} r\left(A\right)=r\left(B\right). \end{array}$$

**Proof**

Set *E* = *A* − *B*, and since ||*B* ^⊕^||_02_ ≠ 0, then, by using Lemma 11, we have ||*E*||_02_ < 1/||*B* ^⊕^||_02_ which implies that

(30) $$\begin{array}{c} r\left(A\right)=r\left(B+E\right)\geq r\left(B\right). \end{array}$$

Since ||*A* ^⊕^||_02_ ≠ 0, then we also have ||*E*||_2_ < 1/||*A* ^⊕^||_02_ which implies that

(31) $$\begin{array}{c} r\left(B\right)=r\left(A-E\right)\geq r\left(A\right). \end{array}$$

Now, the result follows by using (30) and (31).

If *A* ∈ *M*_*m*,*n*_ and *λ*_0_ is the largest eigenvalue of *A* ^~^ *A*, then ${||A||}_{02}=\sqrt{\lambda_{0}}$ in the Minkowski space *μ*. Here, if *P* is the *μ*-symmetric projector, then it is easy to show that *P* ^⊕^ = *P*. Since the eigenvalues of a projector are only 0 and 1, then we have ||*P*||_02_ = ||*P* ^⊕^||_02_ = 1, and by applying Corollary 12, we get the following result.

**Corollary 13**

Let *P* and *Q* be two *μ*-symmetric projectors such that ||*P*−*Q*||_02_ < 1. Then,

(32) $$\begin{array}{c} r\left(P\right)=r\left(Q\right). \end{array}$$

**Theorem 14**

The matrix *A* ∈ *M*_*m*,*n*_ can be written by using SVD as in the form *A* = USV* with U*U = *I*_*m*_, V*V = *I*_*n*_, and *S* is a diagonal if and only if the following conditions hold:

- *σ*(*A* ^~^ *A*) are nonnegative real numbers,
- *A* ^~^ *A* is diagonalizable,
- *N*(*A* ^~^ *A*) = *N*(*A*).

If only assumption (i) is violated but (ii) and (iii) hold of Theorem 14, then we can still get singular value decomposition (SVD). But in the Minkowski space, each of the assumptions can fail even if the other two hold. This is illustrated by the following three counterexamples [18].

**Example 15**

Let $A=\left[\begin{array}{cc} -1 & 1\\ 1 & 1 \end{array}\right]$. Then, $A^{\sim }=\left[\begin{array}{cc} -1 & -1\\ -1 & -1 \end{array}\right]$, and $AA^{\sim }=\left[\begin{array}{cc} 0 & -2\\ 2 & 0 \end{array}\right]$. Hence, *σ*(*A* ^~^ *A*) = {±2*i*} which are not real numbers.

**Example 16**

Let $A=\left[\begin{array}{cc} 1.5 & 1\\ 0.5 & 1 \end{array}\right]$. Then, $A^{\sim }=\left[\begin{array}{cc} 1.5 & -0.5\\ -1 & 1 \end{array}\right]$, and $AA^{\sim }=\left[\begin{array}{cc} 2 & 1\\ -1 & 0 \end{array}\right]$. Hence, *σ*(*A* ^~^ *A*) = {1} and cannot be diagonalized.

**Example 17**

Let $A=\left[\begin{array}{cc} 1 & -1\\ -1 & 1 \end{array}\right]$. Then, $A^{\sim }=\left[\begin{array}{cc} 1 & 1\\ 1 & 1 \end{array}\right]$, and $AA^{\sim }=\left[\begin{array}{cc} 0 & 0\\ 0 & 0 \end{array}\right]$. Hence, *N*(*A* ^~^ *A*) ≠ *N*(*A*).

The following result gives the equivalent conditions for the continuity to be held for the Minkowski inverse of any rectangular matrix.

**Theorem 18**

Let (*A*_*n*_)_*n*∈*ℕ*_ ∈ *M*_*m*,*p*_ be a sequence of matrices such that lim⁡_*n*→*∞*_ *A*_*n*_ = *A*. Then, lim⁡_*n*→*∞*_ *A*_*n*_^⊕^ = *A* ^⊕^ = (lim⁡_*n*→*∞*_ *A*_*n*_)^⊕^ if and only if *r*(*A*_*n*_) = *r*(*A*).

**Proof**

Suppose that lim⁡_*n*→*∞*_ *A*_*n*_^⊕^ = *A* ^⊕^ = (lim⁡_*n*→*∞*_ *A*_*n*_)^⊕^, and set *A*_*n*_ = *A* + *E*_*n*_  such that lim⁡_*n*→*∞*_ *E*_*n*_ = 0 and lim⁡_*n*→*∞*_(*A*+*E*_*n*_)^⊕^ = *A* ^⊕^. Then,

(33) $$\begin{array}{c} \underset{n\to \infty }{\lim}\left(A+E_{n}\right){\left(A+E_{n}\right)}^{⊕}=AA^{⊕}, \end{array}$$

which means that there exists *n*_0_ such that, for any *n* ≥ *n*_0_,

(34) $$\begin{array}{c} {||\left(A+E_{n}\right){\left(A+E_{n}\right)}^{⊕}-AA^{⊕}||}_{02}<1. \end{array}$$

Since (*A* + *E*_*n*_)(*A*+*E*_*n*_)^⊕^ and *AA* ^⊕^ are *μ*-symmetric projectors, then, by Corollary 13, we have

(35) $$\begin{array}{c} r\left(\left(A+E_{n}\right)\right)=r\left(\left(A+E_{n}\right){\left(A+E_{n}\right)}^{⊕}\right)=r\left(AA^{⊕}\right)=r\left(A\right). \end{array}$$

Conversely, suppose that *A* satisfies the SVD conditions as in Theorem 14; then, $B=U^{*}AV=\left[\begin{array}{cc} D_{r} & 0\\ 0 & 0 \end{array}\right]$, where *D*_*r*_ is a diagonal matrix and *U* and *V* are unitary matrices. Suppose also that lim⁡_*n*→*∞*_ *E*_*n*_ = 0 and *r*(*A* + *E*_*n*_) = *r*(*A*) = *r* for any *n* ≥ *n*_0_. Now, set

(36) $$\begin{array}{c} F_{n}=U^{*}E_{n}V=\left[\begin{array}{cc} F_{11}^{\left(n\right)} & F_{12}^{\left(n\right)}\\ F_{21}^{\left(n\right)} & F_{22}^{\left(n\right)} \end{array}\right]. \end{array}$$

Then,

(37) $$\begin{array}{c} r\left(B+F_{n}\right)=r\left(A+E_{n}\right)=r,\;\;B^{⊕}=V^{\sim }A^{⊕}{\left(U^{*}\right)}^{\sim },\\ {\left(B+F_{n}\right)}^{⊕}=V^{\sim }{\left(A+E_{n}\right)}^{⊕}{\left(U^{*}\right)}^{\sim }. \end{array}$$

Since lim⁡_*n*→*∞*_ *F*_*n*_ = 0, then, for ||*D*_*r*_^−1^||_02_ ≠ 0 and ||*A* ^⊕^||_02_ ≠ 0, we have

(38) $$\begin{array}{c} {||F_{n}||}_{02}<\frac{1}{{||D_{r}^{-1}||}_{02}}=\frac{1}{{||A^{⊕}||}_{02}}. \end{array}$$

But ||*F*_*n*_||_02_ ≥ sup⁡_*i*,*j*_{||*F*_*ij*_^(*n*)^||_02_}, and then, by Lemma 11, we have

(39) $$\begin{array}{c} r\left(D_{r}+F_{11}^{\left(n\right)}\right)\geq r. \end{array}$$

On the other hand,

(40) $$\begin{array}{c} r=r\left(B+F_{n}\right)\geq r\left(D_{r}+F_{11}^{\left(n\right)}\right)\geq r. \end{array}$$

Now, by using (39) and (40), we get that *r*(*D*_*r*_ + *F*_11_^(*n*)^) = *r*, which means that (*D*_*r*_+*F*_11_^(*n*)^)^−1^ exists. Also by using the Schur complement, we have

(41) (B+Fn)=[Dr+F11(n)F12(n)F21(n)F21(n)(Dr+F11(n))−1F12(n)]=[IrM](Dr+F11(n))[IrN],

where *M* = *F*_21_^(*n*)^(*D*_*r*_+*F*_11_^(*n*)^)^−1^ and *N* = (*D*_*r*_+*F*_11_^(*n*)^)^−1^ *F*_12_^(*n*)^. Now, if *M* ^~^ *M* ≠ −*I*_*r*_ and *NN* ^~^ ≠ −*I*_*r*_, then, by Definition 2, we can see that

(42) (B+Fn)⊕=[IrN~]((Ir+M~M)(Dr+F11(n))(Ir+NN~))−1 ×[IrM~]

is the Minkowski inverse of *B* + *F*_*n*_. Since lim⁡_*n*→*∞*_ *F*_*n*_ = 0, then lim⁡_*n*→*∞*_ *F*_11_^(*n*)^ = lim⁡_*n*→*∞*_ *F*_21_^(*n*)^ = lim⁡_*n*→*∞*_ *F*_12_^(*n*)^ = 0. Therefore, lim⁡_*n*→*∞*_(*D*_*r*_+*F*_11_^(*n*)^)^−1^ = *D*_*r*_, lim⁡_*n*→*∞*_ *M* = 0 = lim⁡_*n*→*∞*_ *M* ^~^, and lim⁡_*n*→*∞*_ *N* = 0 = lim⁡_*n*→*∞*_ *N* ^~^. Now, by using the fact that the map which transforms an invertible matrix to its inverse is continuous, consequently, we find that

(43) $$\begin{array}{c} \underset{n\to \infty }{\lim}{\left(B+F_{n}\right)}^{⊕}=\left[\begin{array}{c} I_{r}\\ 0 \end{array}\right]D_{r}^{-1}\left[\begin{array}{cc} I_{r} & 0 \end{array}\right]=\left[\begin{array}{cc} D_{r}^{-1} & 0\\ 0 & 0 \end{array}\right]=B^{⊕}, \end{array}$$

which completes the proof of Theorem 18.

## 5. Conclusion

Several attractive properties and conditions of the Minkowski inverse *A* ^⊕^ in the Minkowski space *μ* are presented. In our opinion, it is worth extending these properties and establishing some necessary and sufficient conditions for the reverse order rule of the weighted Minkowski inverse *A*_*M*,*N*_^⊕^ in the Minkowski space *μ* of two and multiple matrix products.
